# Breakfast Beverages

**Published:** 1883-11

**Authors:** 


### Breakfast Beverages.

The following practical remarks we take from the *Brit. Med. Jour.*:

Each of our commoner breakfast beverages, namely, tea, coffee, and cocoa, presents sundry relative advantages and disadvantages, which have been well established by scientific experiments and general experience, and which are qualities that sometimes assume a special importance in certain conditions of health, habit, occupation, climate, and disease. Warm infusion of tea has been proved to have a marked stimulant and restorative action upon the brain and nervous system, and this effect is not followed by any secondary depression. It further increases the action of the skin, and raises the number of the pulse, while it has but little effect upon urination, excepting simply as a watery diuretic. It tends to lessen the action of the bowels. Dr. Parkes found that tea is most useful as an article of diet for soldiers. The hot infusion is a potent protective against extremes both of heat and of cold; and Sir Ranald Martin proved it to be particularly valuable in great fatigue, especially in hot climates. Coffee, like tea, when used as an article of diet, especially affects the nervous system. It is a brain and nerve stimulant; in very large doses it produces tremors. It increases the action of the skin, and it appears to have a special power in augmenting the urinary water. It increases both the force and frequency of the pulse. Unlike tea, it tends to increase the action of the bowels. Coffee has been proved to be an important article in a soldier's dietary, as a stimulant and restorative. Like tea, it acts as a nerve-excitant, without producing subsequent depression. It is serviceable against excessive variations of cold and heat, and its efficacy in these respects has been established in antarctic expeditions, as well as in India and other hot climates. Dr. Parkes pointed out that coffee has a special recommendation in its protective influence against malaria. While admitting that the evidence on this point was not strong, he held it to be insufficient to authorize the large use of coffee in malarious districts. Coffee should be used as an infusion. If coffee be boiled, its delicate aroma is dissipated. The theobromin of cocoa is, chemically, identical with the thein of tea, and the caffein of coffee. While tea and coffee are comparatively valueless as true food, cocoa, by reason of the large quantity of fatty and albuminoid substances it contains, is very nourishing, and is of high dietetic value as a tissue-forming food. Compared with tea and coffee, it is a food rather than a stimulant, being akin to milk in its composition and place in the diet-scale. It is useful to sustain the weakly and to support the strong in great exertion, as a readily assimilable and general form of nourishment.

